# Integrating sequence and structural biology with DAS

**DOI:** 10.1186/1471-2105-8-333

**Published:** 2007-09-12

**Authors:** Andreas Prlić, Thomas A Down, Eugene Kulesha, Robert D Finn, Andreas Kähäri, Tim JP Hubbard

**Affiliations:** 1 The Wellcome Trust Sanger Institute, Hinxton, Cambridge, UK; 2 European Bioinformatics Institute, Hinxton, Cambridge, UK; 3 Wellcome Trust/Cancer Research UK Gurdon Institute, Cambridge University, Cambridge, UK

## Abstract

**Background:**

The Distributed Annotation System (DAS) is a network protocol for exchanging biological data. It is frequently used to share annotations of genomes and protein sequence.

**Results:**

Here we present several extensions to the current DAS 1.5 protocol. These provide new commands to share alignments, three dimensional molecular structure data, add the possibility for registration and discovery of DAS servers, and provide a convention how to provide different types of data plots. We present examples of web sites and applications that use the new extensions. We operate a public registry of DAS sources, which now includes entries for more than 250 distinct sources.

**Conclusion:**

Our DAS extensions are essential for the management of the growing number of services and exchange of diverse biological data sets. In addition the extensions allow new types of applications to be developed and scientific questions to be addressed. The registry of DAS sources is available at

## Background

New large scale techniques in biology have produced a rapidly increasing range of publically available data. A number of centralized database resources are available that aim to integrate this data, such as Entrez [1], Interpro [2], MSD [3], and Ensembl [4]. Databases that integrate huge datasets are faced with problems regarding scaleability of their storage facilities, how to manage frequent updates and how to exchange the data with the community. Rather than building bigger centralized repositories, an alternative strategy is to encourage individual data providers to offer their results in standard formats, then provide users with tools which can find and communicate with these distributed repositories, and build integrated views on demand. The *Distributed Annotation System *(DAS) is a widely used system for biological sequence annotation that follows this latter approach [5]. DAS is frequently used for

1. Integration of personal data into major bioinformatics portals like Ensembl [4].

2. Integration of the annotations from external sources into local applications.

3. Access to the most recent versions of biological databases without the need for local installations.

### Reference and Annotation servers

DAS is a simple client-server network protocol. DAS clients make requests by fetching a defined URL from a DAS server, and receive simple XML responses. It is a web service, in the sense that all requests are made using the HyperText Transfer Protocol (HTTP). This also allows to use existing web proxy infrastructure for accessing remote servers through firewalls. An important part of the request URL is the DAS command, which specifies the type of information that the client is requesting, and thus the response format which it expects to receive. Key commands in the DAS 1.5 protocol are *sequence*, which returns all or part of a DNA or protein sequence, and *features*, which returns a set of annotated regions on a sequence, such as genes or protein domains. See [6] for the full specification of the DAS 1.5 protocol.

Central to the DAS system is the idea of *reference objects*. These are biological data objects with stable identifiers which are targets for annotation. In the original DAS protocol, the reference objects are always biological sequences: chromosomes, scaffold sequences from genome assemblies or protein sequences. When a DAS client starts, its first action is to connect to an appropriate reference server and retrieve the reference sequence. Once a reference object has been loaded, the DAS client will contact one or more *annotation *servers to obtain the data provided for the reference objects. Typical annotations might include sets of predicted exons on a genome sequence, or matches to a protein domain model on a protein sequence. It is the client's responsibility to collect all relevant annotations and display them in a user-friendly manner. Genomic sequences are generally much longer than protein sequences, in terms of number of nucleotides and amino acids, so it is possible to restrict DAS requests to sub-ranges of the data. Biological sequence data is dynamic: sequences change as new sequence data becomes available. Thus it is important that DAS clients are able to check that the annotation they have received match the available reference sequence. The ideal solution is a version number for each sequence, administered by whatever authority assigns identifiers for sequences that is incremented each time the sequence changes. Not all biological databases provide such a version number though, so the members of the BioSapiens project [7] have agreed to use the MD5 digest of the sequences for both the reference objects and the annotations as part of the version attribute in the DAS responses.

## Results

### New DAS commands

The original DAS specification was rather genome and sequence centric. DAS mediated data exchange and visualisation today is heavily used in the popular genome browsers like Ensembl ([4]), Wormbase [8], and GBrowse [9]. Several protein sequence DAS clients are available like *Dasty *[10], ProView [11] or the CBS DAS Viewer [12]. The multiple sequence alignment viewer Jalview has recently been extended to support accessing features through DAS [13]. CARGO is a flexible system for visualization and analysis of biological information which among several different sources of data can use DAS [14].

To generalize the system and enable the development of new applications, we have extended the specification and added several new commands. The new DAS commands are:

1. *alignment *– request an alignment or mapping between two or more reference objects. The alignment format is quite general, and can specify pairwise and multiple alignments between protein sequences, structures and chromosomes.

2. *structure *– request coordinate data for three dimensional reference objects such as protein structures.

3. *sources *– request and query meta information about available DAS sources.

## Implementation

The guiding principle for the design of the new DAS commands was to provide a *reference *object for three dimensional information, similar to the sequence command. The alignment command should be rather generic and support multiple alignments of objects of the same data type. In connection with the *coordinate systems *introduced below, it can also provide mappings between objects of different data types, like mappings between protein sequence and structure residue positions or alignments of chromosomes. Similar to the original DAS commands, data should be pre-computed. For serving structure alignments, the actual calculation of the superimposition should be done on the server side, so the client can be independent from the actual implementations of structure alignment algorithms and support any of the number of available methods, for example [15-18].

We have extended the two DAS server implementations ProServer, which is written in Perl [19] and its Java counterpart, Dazzle [20] to support the new commands. For a detailed documentation on the specification of the new DAS commands please see [21].

### Publishing and discovery of DAS sources

DAS is now a widely-accepted communication protocol. With a growing number of people offering data via DAS, it has become increasingly difficult to keep track of the different servers and the functionality and data they offer. Originally there was no specification for a registration mechanism and if a user wanted to add a new DAS source to a client, they were expected to discover the server's URL outside of the DAS system, perhaps by reading web pages or papers, then manually enter the URL into their DAS client. Moreover, with the extensions presented here DAS becomes applicable to new data types, which adds another level of complexity. We believe a registration system is essential to support the continued development and usage of DAS.

**Figure 1:**
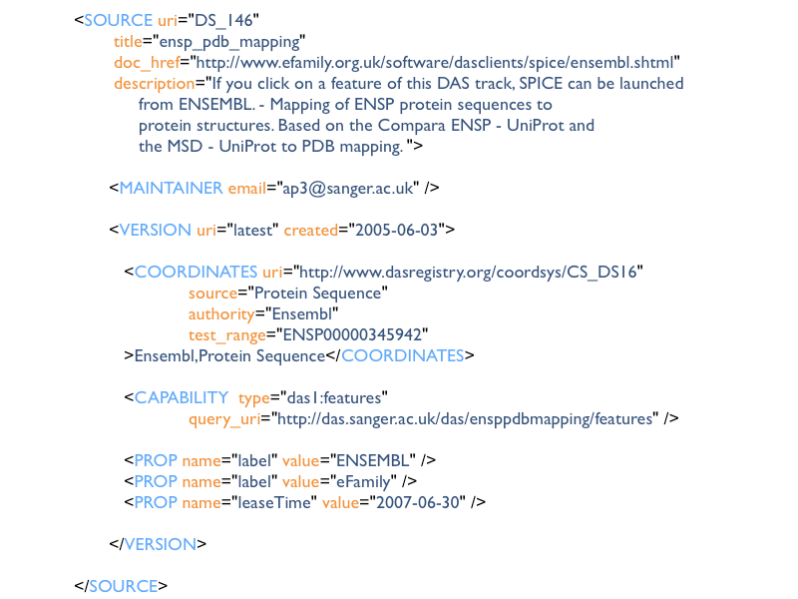
Example of the new DAS sources response. It provides meta description for DAS servers. The meaning of the different elements are: SOURCE: This DAS source provides mappings to protein structures for Ensembl proteins. It has a unique ID in the DAS registration server (DS_146). The doc_href provides a link to a web page with more documentation on this data. MAINTAINER: contact this person if there is a problem with the server, or if you have any questions about it. VERSION: this DAS source has been registered at the DAS registry in 2005 and provides the latest available data. COORDINATES: The data is served as annotations for the set of protein sequences provided by Ensembl. An example accession code is provided which can be used to check if the server is giving a valid response. CAPABILITIES: The DAS/1 features command is being supported by this server. The query uri field provides the location of the DAS source. PROP: Different labels can be assigned. The leaseTime is the last time when this server has been validated successfully.

In order to address this problem we have developed a DAS extension for communicating with registry servers, the *sources *command. Registry entries include the base URL for communicating with a server, a human-readable description of the data it provides, and also some machine-readable meta information which helps clients to automatically decide which sources might be relevant to a given reference object. See Figure 1 for an example how a DAS source is being described with this meta information.

### Multiple Data Types

DAS is used to annotate many different kinds of biological entities. Genome sequences, gene loci, protein sequences, and molecular structures are currently the most common targets for DAS annotation. The DAS registration system therefore needs to be able to deal with the different data types. A DAS client on the other side usually will only support a certain set of these types, for example, a particular view of the Human genome will not necessarily be able to display data that has been annotated on the Chimpanzee genome. To allow data integration, the DAS clients must be able to find all the DAS annotation sources which can be used with a particular reference object. One of the goals of the DAS registration service is to provide a description of these sets of data types. It is called *coordinate system*, and can also be thought of as a "namespace" for reference objects.

There are several parts to the definition of a coordinate system:

• The *authority *or name of a data set. This usually is the name of an institution or organization that defines identifiers or accession codes. For example, UniProt is an authority to assign protein sequence accession codes.

• The *type *of object refers to the physical entity being used. Currently supported are *Chromosome, Clone, Contig, Gene_ID, NT_Contig, Protein Sequence, Protein Structure*, and *Scaffold*.

• The *organism *from which the data was derived. This field can be empty in the case of multi-species databases like UniProt, but is important for genome assemblies.

• Some resources, especially genome assemblies, have a versioning scheme which applies to the entire collection of sequences rather than to a single sequence. In this case, the optional *version *field can represent this, and allows data sources annotating several different versions of a given genome to co-exist in the same registration server.

### Implementation of a DAS Registry

Based on the concepts of the *sources *meta description of DAS sources, we have established a central DAS registry which implements this protocol and fulfils the following roles:

1. It allows discovery of available DAS sources via a web page, or as machine-readable XML which can be used directly by DAS client programs.

2. It automatically validates registered DAS sources to ensure that they return well formed DAS XML.

3. It periodically tests DAS sources and notifies their administrators if they are unavailable.

4. It can group the registered DAS sources according to the coordinate systems of their data.

5. It can also communicate bi-directionally with DAS clients and activate or highlight DAS sources in clients.

The DAS registration server is available at [22]. Currently it contains a listing of more than 250 DAS servers provided by about 40 groups in 13 countries. To obtain a listing of meta information about all registered DAS servers, the new *sources *DAS command is provided at [23]. The same command can be used to batch-upload DAS servers, for example Ensembl provides a listing of DAS sources via [24]. The registry automatically obtains this listing and updates the meta information for these servers.

The DAS registry is backed by a MySQL relational database, and can be accessed in several different ways. As well as the DAS-style *sources *interface, a HTML front-end is provided which allows manual interaction with the registry [22]. A SOAP style webservice can be accessed from [25].

Two tools that the DAS registry offers are validation and auto-activation of DAS sources in DAS clients.

#### Validation

There are a number of different DAS server programs available. Frequently used server libraries include Dazzle [20], ProServer [26], LDAS [27] or GBrowse [28]. Due to the simplicity of DAS, sometimes individually implemented CGI scripts are used as well, thus it is important to ensure that a correct response is given regardless of the implementation. To do this the DAS registry employs a validation mechanism such that only servers providing well formed DAS-XML can get registered. For this validation a *test code *is required, which is an accession code that identifies a reference object for which features are provided. To ensure that the listing within the DAS registry is current, the servers are periodically validated to ensure that the server is still available and the XML response is well formed. Successful attempts are logged, and a graphical summary of the availability of a DAS source is provided via the registry's web interface. If the DAS source can not be validated for more than two days, a *watchdog *can optionally inform the server's administrator by email. If a server is down for a longer period of time, the server administrator can be contacted to inquire about the status of the server. If the server remains unavailable for an extended period, it will be removed from the listing.

#### Auto-activation

Users can browse and search through the DAS registry website. If they find an interesting DAS source it can be conveniently be enabled or highlighted in their favorite DAS client, if this client supports this. The listing of each DAS server in the registry's web interface contains specially formatted links to the clients. When selected, these links not only launch the DAS client, but highlight or turn on that particular DAS source, which we have termed auto-activation. At present, the clients that support the registry directed auto-activation are Ensembl [4], SPICE [29], and Dasty [10]. The registry also provides a *send to friend *mechanism to share auto-activation links by email.

### Positional and non-positional features and plots of data

The DAS protocol provides the *features *command to share annotations that have a *start *and *stop *position. This convention does not deal well with special cases where annotation can not be assigned to a defined region, or where the feature has a size of one. For data that is *non-positional*, such as literature references, the working convention has been adopted of providing 0 as the start and stop positions.

**Figure 2:**
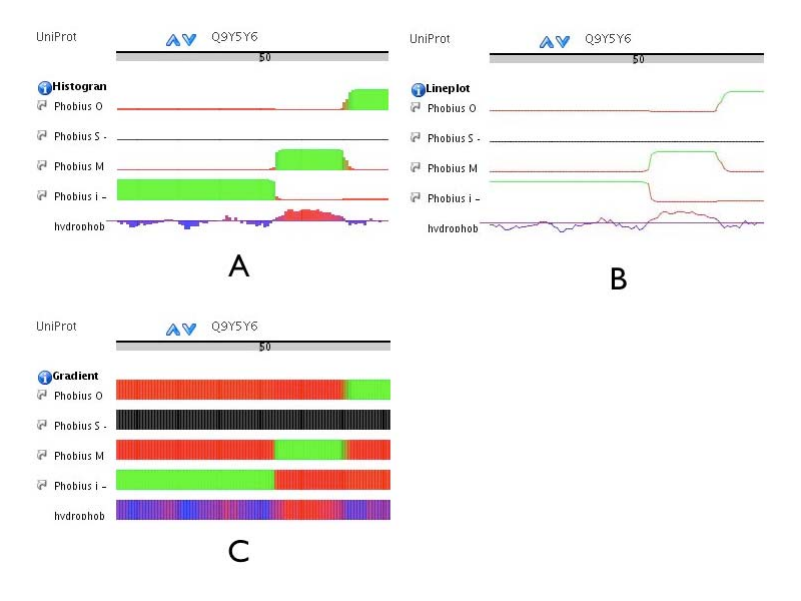
We are providing a convention how to configure the display of different data-plots via the DAS stylesheet command. Here an example how this is used for plotting the scores of the signal peptide and transmembrane topology predictor Phobius [48], as well as hydropathicity data for the Suppressor of tumorigenicity protein 14 (Q9Y5Y6). The DAS features used in the three displays are exactly the same. The configuration is obtained from the stylesheet responses. The example can be also viewed via [49]. A) Using the stylesheet for histogram display. B) Using the stylesheet for lineplot display. C) Using the stylesheet for gradient display.

Some data sets, such as hydropathicity plots or signal peptide prediction scores, can be transmitted via DAS by providing a different score for each feature position. The DAS 1.5 specification provides the *stylesheet *command which allows to specify how a DAS client should visualize data. We developed a convention how to configure different types of plots for Ensembl and SPICE [30] and implemented support for this in the two DAS clients (see Figure 2).

### Applications

The new DAS extensions presented here are actively being used by several web sites and applications. This section highlights a couple of these as examples.

#### Pfam

**Figure 3:**
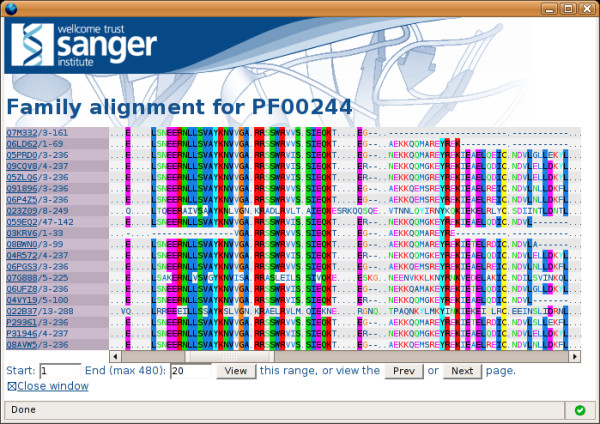
The Pfam DAS alignment viewer. Here, the first 20 sequences from the 14-3-3 [Pfam:PF00244] full alignment are requested from the pfamFullAlign DAS alignment server and displayed. The alignment is coloured according to the consensus of the whole alignment, which is provided as a DAS feature. The client allows simple paging through an alignment using the navigation buttons below the alignment.

The *Pfam *database [31] now makes a large proportion of its data available via DAS. In addition to the classical DAS-*feature *command that is used to provide domain annotations of individual protein sequences, the Pfam multiple sequence alignments are also made available via DAS (using the alignment extension described above). One of the options in the specification allows the retrieval of one or more ranges from the requested query alignment. This option is of particular use when trying to view the very big Pfam alignments. For example, the Pkinase [Pfam:PF00069] *full *alignment contains 19113 sequences (Pfam release 21.0) making it, at best, very slow to visualise the alignment when loaded as a single file. By requesting ranges, the alignment visualisation is much faster. However, most alignment visualisation tools utilise some kind of colouring scheme to highlight regions of conservation based on the properties of the whole alignment. Therefore, to enable a region of an alignment to be placed in context of the whole alignment, Pfam also provides the alignment consensus via a DAS-*feature *request. The data from these two requests can be combined such that requested alignment regions can be coloured according to similarity (see Figure 3).

#### Mapping of Genome Features on Protein structures

We have set up alignment DAS server that provide mappings from the Ensembl peptide sequences to Uniprot, and from Uniprot to PDB. The DAS client *SPICE*, is a browser of protein structures, sequences, and their annotations [29]. Based on the alignment data sources we extended SPICE, which now can easily show the location of genomic features, like SNPs or Intron/Exon boundaries mapped onto the three dimensional structure.

#### Multiple Structure Alignments

**Figure 4:**
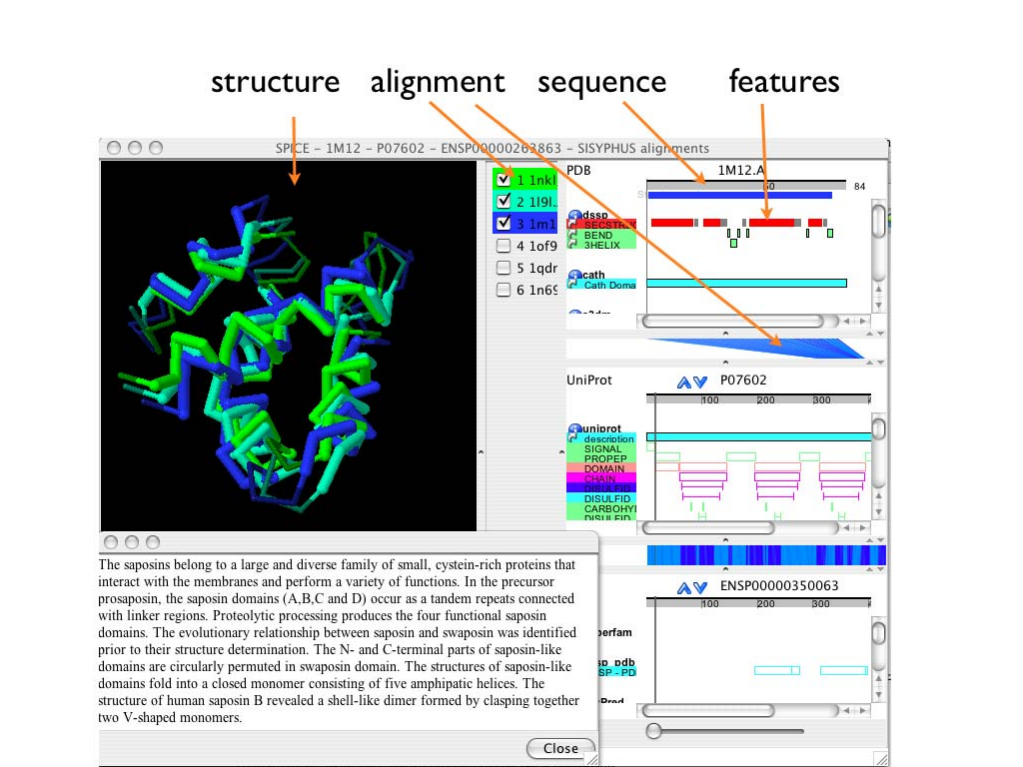
SPICE displays a multiple structure alignment of the Saposins, as provided by the SISYPHUS database. It is possible to choose which of the structures should be superimposed and sequence features can be mapped on the three dimensional alignment. The top of the image shows the different DAS commands that are being used in order to obtain the integration of the data.

The alignment commands can be utilized to export pairwise and multiple protein structure alignments, a feature which has been used by the SISYPHUS database. SISYPHUS is a collection of proteins with non-trivial structural relationships and their alignments [32]. It provides a large amount of the data via DAS. The SISYPHUS multiple structure alignments can now be visualized using SPICE which has been recently extended to view multiple protein structure alignments (see Figure 4, [33]).

Another application of the new DAS structure and alignment commands was the CASP experiment [34], which has the goal to independently assess protein structure predictions. To do so, information about structures that have not been released to the public is passed to the structure prediction teams. These teams then provide models of the "unknown" structure.

To make evaluation and assessment of the predictions easier and more open, alignments of the predictions against the target were made available using DAS and visualized using SPICE. The different structures can be compared with each other and it is possible to switch between sequence dependent and independent alignment servers [35,36].

## Discussion

DAS is an important system for exchanging and viewing biological data. The simple, but powerful original design of the DAS specification allowed scope for extension of the protocol. Here we presented several extensions to the core DAS specification that allow to manage, publish and discover the ever growing number of DAS sources and to deal with alignments and protein structure data, hence allowing new types of applications built on DAS. We envisage further extensions to the DAS protocol in the future. A new extension for electron microscopy data has recently been published by Macías et al. [37]. DAS has also received attention beyond the biological community and now even serves as a model in Astrophysics [38]. DAS has many similarities to webservices that are provided using the Simple Object Access Protocol (SOAP). SOAP based services have been made available by a number of institutions, e.g. [39-42]. They use XML requests and responses for the transport of information. In contrast to DAS there is no default definition how the transported information should look like, although there are approaches to provide standards for some data types [43]. BioMoby is a system for interoperability between data providers, analysis services providers, and client implementations which is using SOAP as an envelope [44].

In contrast to SOAP based web services, DAS follows the paradigm of REST, the Representational State Transfer [45]. The basic difference is that DAS does not have an XML envelope for its messages, but uses well defined URLs for the DAS commands. The DAS responses are transported as XML documents. SOAP is frequently used to request remote calculation of data. In contrast the data provided with DAS is most of the time pre-calculated or can be computed quickly. Together with the Taverna project [46] we agreed on the addition of a "come back later" response for DAS which will allow to perform longer lasting computations triggered by DAS requests. This can be used to establish a bridge between DAS and Taverna workflows. A prototype implementation for this is available via the Dasobert Java library.

A concept sometimes used in connection with SOAP based webservice is the *Service Oriented Architecture *(SOA). A SOA consists of three components. A service provider, a consumer and a service registry. This concept can also be applied to DAS, with DAS servers being the service providers and DAS clients the consumers. By providing a registration server, we have extended DAS to become a full SOA.

Communication with the registry has been integrated into several web sites and applications. Ensembl, Pfam, Dasty, Jalview and SPICE now all can talk to the registry and users can access the registered DAS servers through different configuration interfaces.

Currently the version 2.0 of DAS specification is emerging [47]. We are actively participating in this development process. The *sources *specification for describing DAS services has been adopted as part of the core DAS/2 specification. DAS/2 is being defined with extensibility in mind and will allow the addition of protocols for sharing structure and alignment information.

## Conclusion

The Distributed Annotation System is becoming widely accepted demonstrated by the fact that support for it is being built into more and more web sites and applications. Here we have presented several extensions that help the DAS system to scale, add support for new data types, and allow the development of new applications. Using these new DAS commands it has become possible to seamlessly walk from the genome level to the protein structure level and to share sequence and structure alignments.

## Availability and Requirements

• Project home page: 

• Operating system(s): Platform independent

• Programming language: Java, Perl

• Other requirements: Internet Browser

• License: LGPL

• Any restrictions to use by non-academics: none

## Authors' contributions

AP implemented SPICE and the DAS registration server, extended the Dazzle server and wrote the paper. TD contributed several libraries, was involved in the design and planning of this project, is main author of the Dazzle server and helped with writing the paper. EK extended the Ensembl code base with new DAS functionality. RF is responsible for the Pfam website, for extending ProServer to support the new DAS commands and contributed some paragraphs to the paper. AP, TD, RF, TH designed the DAS extensions. AP, TD, RF, EK, AK set up and maintain various DAS servers. TH provided substantial advice and guidance during all phases of the project. All authors read and approved the final manuscript.
